# Aortopulmonary Window Associated with an Ascending Aorta Aneurysm in an Adult 

**Published:** 2015-07-03

**Authors:** Rachid El Haouati, Yassine Boukaidi, Zahira Zouizra, Drissi Boumzebra

**Affiliations:** *Cardiovascular Surgery Unit, Mohamed VI Universitary Hospital, Marrakech, Morocco.*

**Keywords:** *Aortopulmonary septal defect*, *Aneurysm*, *Aorta*, *Heart defects*, *congenital*

## Abstract

Aortopulmonary window (APW) is a rare congenital malformation. It results from an incomplete division between the ascending aorta and the pulmonary artery. We describe a 26-year-old male, who presented with a grade II exertional dyspnea and palpitations. Echocardiography revealed an APW with an ascending aorta aneurysm. He underwent surgery under cardiopulmonary bypass without aortic cross-clamping. The APW was closed via the pulmonary artery flap technique using an autologous pericardial patch, and the aneurysm was repaired through the reduction aortoplasty technique. The patient was discharged on the 4^th^ postoperative day. At 2 years' follow-up, he had remained asymptomatic and echocardiography showed aortic valve competence, ascending aorta diameter of 38 mm, and no residual shunt.

## Introduction

The aortopulmonary window (APW) is a rare congenital malformation and represents only 0.1% of all congenital heart diseases.^1^ It results from an incomplete division between the ascending aorta and the pulmonary artery. The natural history of this anomaly is characterized by the early manifestations of congestive heart failure, gradual development of irreversible pulmonary hypertension occurring early in life, and death if not treated.^2^^-^^4^ Surgical correction is, therefore, advised as early as possible. Although there are various reports of an uncorrected APW in adulthood,^2^^, ^^4^ there are very few reports of operated cases. We describe a 26-year-old male patient suffering from an APW associated with an ascending aorta aneurysm who underwent successful surgery and discuss the clinical and surgical features of this condition.

## Case Report

**Figure 1:**
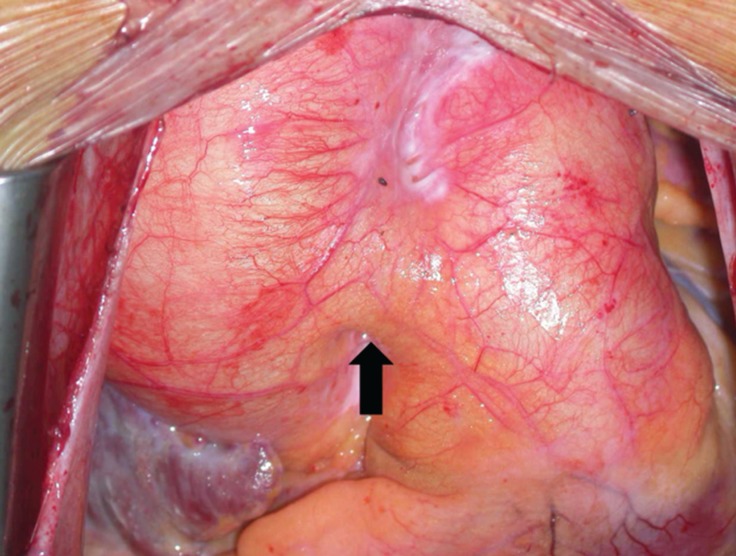
Preoperative finding: aortopulmonary window (black arrow)

A 26-year-old man was admitted to our institution for the assessment of a grade II exertional dyspnea and palpitations. The patient was born from a first-degree consanguineous marriage. He had no history of recurrent respiratory infections. On physical examination, he had normal vital signs: blood pressure at 140/50 mmHg with a bounding pulse and saturation at 96% on room air. He had pectus carinatum (Figure 1). Auscultation found a 4/6 left laterosternal systolic murmur. There were no signs of heart failure. Electrocardiography showed regular sinus rhythm at 80 beats/minute with left heart axis deviation and negative anteroseptal T waves. Chest X-ray demonstrated marked pulmonary vascularity and cardiomegaly. Echocardiography revealed a dilated left ventricle with a good ejection fraction. The ascending aorta was enlarged above the sinotubular junction measuring 48 mm, with trivial aortic regurgitation. There was no abnormality in the coronary arteries. A 12-mm APW was identified with a left-to-right shunt. The surgical closure of the APW in conjunction with reduction aortoplasty was indicated.

**Figure 2:**
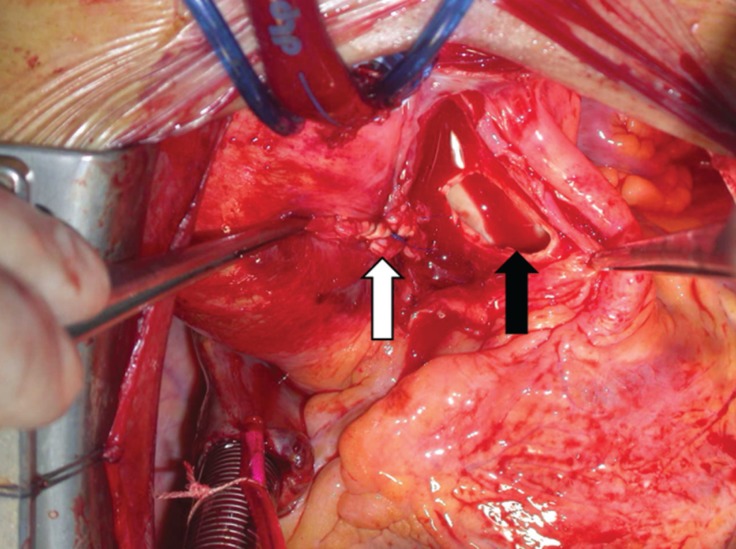
Closure of the aortic side of the window with the parietal flap resected from the main pulmonary artery (white arrow). Residual defect of the main pulmonary artery (black arrow)

**Figure 3:**
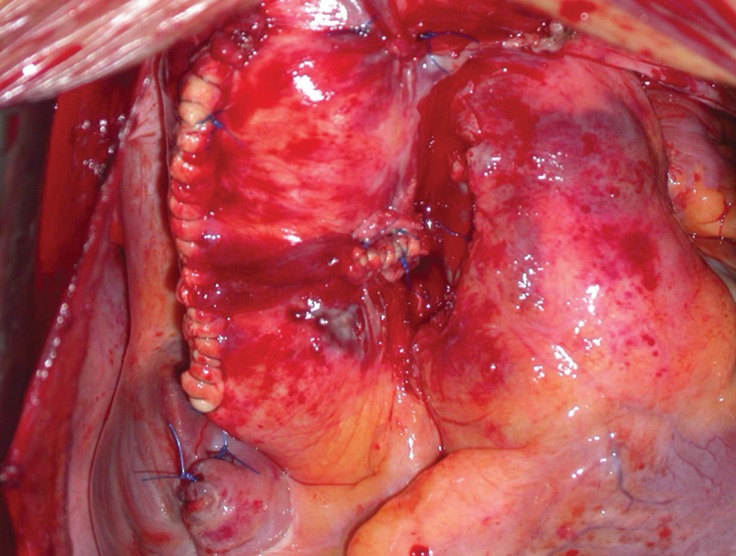
Surgical result at the end of the procedure

The patient underwent surgery through median sternotomy. The dilatation of the ascending aorta and the APW were plainly evident. Surgery was conducted under normothermic cardiopulmonary bypass between bicaval cannulas and an aortic cannula without aortic-cross clamping. After a lateral clamping of the ascending aorta excluding the APW, without compromising the aortic flow, the pulmonary artery was discharged and then incised a few millimeters to the left of the APW to create a flap (Figure 2). This flap was thereafter used to reconstruct the ascending aorta with a continuous 5-0 polypropylene suture. The main pulmonary artery defect was closed using glutaraldehyde-treated autologous pericardium. The aortic dilatation was repaired after repositioning of the clamp to the anterior side of the aorta. A longitudinal oval resection of the aortic wall was subsequently performed. The aortotomy was closed in two rows using polypropylene 4-0 suture (Figure 3).

The patient's postoperative course was uneventful, and he was discharged on the fourth postoperative day. At 2 years' follow-up, he had remained asymptomatic and echocardiography showed aortic valve competence, ascending aorta diameter at 38 mm, and no residual shunt.

## Discussion

The APW is a rare congenital heart anomaly and appears in approximately 0.1% of all congenital heart diseases.^1^ In this anomaly, a communication exists between the ascending aorta and the pulmonary trunk. The APW may be located anywhere from above the semilunar valves to the more distal ascending aorta. Despite the rarity of the APW, several classifications have been proposed. The latest and the most commonly used one was described by Jacobs et al.,^1^ who classified the condition as proximal, distal, total, and intermediate, depending on the site in relation to the pulmonary artery. Most commonly, the APW is an isolated single defect.^5^ Anomalies such as interrupted aortic arch, coarctation, transposition of the great arteries, and tetralogy of Fallot have been reported in association with the APW.^6^^-^^8^ Our patient had an intermediate type of the APW; and to our knowledge, it is the first reported case of the APW with an ascending aorta aneurysm.

The development of the aneurysm can be explained in part by the turbulent flow in the ascending aorta; this turbulence is a result of several mechanisms, including the wide pulse pressure (it reached 90 mmHg in our patient), the directional changes in the flow in the adjacent area of the APW, and the high left ventricular output.^9^

The prognosis of an uncorrected APW in infancy is poor with a high mortality in the first year of life. Adult patients with this anomaly usually present with congestive heart failure or irreversible pulmonary hypertension, contraindicating surgical repair. Indeed, only a few adults with a successfully operated APW have been reported in the literature.^2^^, ^^4^^, ^^5^

The clinical presentation of patients with the APW is similar to that of other patients with large left-to-right shunts. The diagnosis of the APW can be confirmed with transthoracic echocardiography. The communication between the aorta and the pulmonary artery can be best visualized and sized in the high parasternal short-axis view. This noninvasive investigation allows the exact localization of the defect and demonstrates its size.^4^^, ^^5^ Cardiac catheterization is rarely indicated and reserved for patients at risk of elevated pulmonary vascular resistance or any patient in whom the anatomy cannot be adequately defined by echocardiography.^1^^, ^^5^

Surgical repair is indicated immediately after the diagnosis of the APW is established, regardless of the patient’s age. Although early surgical repair is recommened,^3^^, ^^6^^, ^^10^ some patients may survive until adulthood without developing irreversible pulmonary vascular hypertension.^2^^-^^5^ These patients could benefit from surgery with good outcomes. The surgical treatment of the APW represents a continuous evolution of technique. Since the first successful ligation was performed by Gross in 1948,^11^ several surgical techniques have been proposed from simple ligation to closure of the defect with a pulmonary artery flap. The closure of the APW can be performed off-pump, avoiding the morbidity associated with cardiopulmonary bypass, but with greater risk of leaving a residual shunt. Transaortic repair with patch closure is the most widely used technique. 

The technique that we chose in our patient precluded cardiac ischemia. Our use of an autologous pulmonary patch allowed aortic defect closure with lesser risk of embolic complications and aortic valve distortion.^12^ We repaired the ascending aorta aneurysm using reduction aortoplasty rather than graft replacement. There is a general agreement that patients with an ascending aorta aneurysm with a diameter exceeding 60 mm should have ascending aorta replacement. We believe that aortoplasty of the ascending aorta has an advantage over graft replacement in surgical invasiveness and risk of complications related to the graft (e.g. infection and thromboembolism). Nevertheless, this procedure is applicable only to selected patients with an appropriate sinus of Valsalva diameter and a normal aortic wall structure.

In the current era, early mortality following the repair of a simple APW approaches zero and the long-term outcome should be excellent.^3^ Early morbidity includes pulmonary artery stenosis, residual shunt, and aortic valve distortion. Therefore, long-term follow-up is mandatory.^3^^, ^^10^

## Conclusion

In addition to the complications stated in the literature, the evolution of an untreated APW in infancy may lead to the ascending aorta enlargement: this particular condition should be managed with conservative technique.

## Notes:

***This paper should be cited as:*** El Haouati R, Boukaidi Y, Zouizra Z, Boumzebra D. Aortopulmonary Window Associated with an Ascending Aorta Aneurysm in an Adult. J Teh Univ Heart Ctr 2015;10(3):156-158.
